# Sleep experiences and longitudinal brain changes in cognitively normal older adults

**DOI:** 10.1002/alz.088817

**Published:** 2025-01-03

**Authors:** Jung‐Min Choi, Min Soo Byun, Dahyun Yi, Joon Hyung Jung, Bo Kyung Sohn, Gijung Jung, Hyejin Ahn, Jun‐Young Lee, Yun‐Sang Lee, Yu Kyeong Kim, Dong Young Lee, KBASE Research Group

**Affiliations:** ^1^ Department of Neuropsychiatry, Seoul National University Hospital, Seoul Korea, Republic of (South); ^2^ Department of Psychiatry, Seoul National University College of Medicine, Seoul Korea, Republic of (South); ^3^ Institute of Human Behavioral Medicine, Medical Research Center, Seoul National University, Seoul Korea, Republic of (South); ^4^ Chungbuk National University Hospital, Cheongju Korea, Republic of (South); ^5^ Sanggye Paik Hospital, Inje University College of Medicine, Seoul Korea, Republic of (South); ^6^ Interdisciplinary program of cognitive science, Seoul National University, Seoul Korea, Republic of (South); ^7^ Department of Neuropsychiatry, SMG‐SNU Boramae Medical Center, Seoul Korea, Republic of (South); ^8^ Department of Nuclear Medicine, Seoul National University College of Medicine, Seoul Korea, Republic of (South); ^9^ Department of Nuclear Medicine, SMG‐SNU Boramae Medical Center, Seoul Korea, Republic of (South)

## Abstract

**Background:**

Disturbed sleep has been associated with Alzheimer’s disease (AD) dementia. Nevertheless, limited information is yet available for the relationship between sleep experiences and longitudinal changes of brain pathologies related to AD. This study aimed to investigate the association between late‐life sleep experiences and the prospective changes of *in vivo* AD pathologies and cerebrovascular injury over 2 years in cognitively normal (CN) old adults.

**Method:**

A total of 225 CN older adults who participated in the Korean Brain Aging Study for the Early Diagnosis and Prediction of Alzheimer’s Disease (KBASE) study between 55 and 90 years of age were included. All participants were also systematically interviewed using a structured interview form to determine sleep duration and subjective sleep quality at baseline. All of them underwent a comprehensive clinical assessment, [^11^C] Pittsburgh Compound B positron emission tomography (PET), [^18^F]fluorodeoxyglucose (FDG)‐PET, and magnetic resonance imaging at baseline and 2‐year follow‐up. A subset of participants (n = 44) also underwent [^18^F]AV‐1451 PET at baseline and 2‐year follow‐up. All analyses were adjusted for age, gender, education, apolipoprotein E ε4 status, vascular risk score, Hamilton Depression Rating Scale score, and use of sleep medication.

**Result:**

Poor sleep quality at baseline was significantly associated with both higher baseline global Aβ retention and its longitudinal increase over 2 years, but not with other AD neuroimaging markers including tau deposition, AD‐related neurodegeneration, and white matter hyperintensity (WMH) volume. There was no significant relationship of sleep duration with any AD neuroimaging markers and WMH volume.

**Conclusion:**

Our findings suggest that poor sleep quality in late life may predict or contribute to prospective increase of brain amyloid deposition in the near future.

